# (*E*)-4-Hy­droxy-*N*′-(2-meth­oxy­benzyl­idene)benzohydrazide

**DOI:** 10.1107/S1600536812021952

**Published:** 2012-05-23

**Authors:** Hesham Hussein Rassem, Abdussalam Salhin, Baharuddin Bin Salleh, Mohd Mustaqim Rosli, Hoong-Kun Fun

**Affiliations:** a School of Chemical Sciences, Universiti Sains Malaysia, 11800 USM, Penang, Malaysia; b School of Biological Sciences, Universiti Sains Malaysia, 11800 USM, Penang, Malaysia; c X-ray Crystallography Unit, School of Physics, Universiti Sains Malaysia, 11800 USM, Penang, Malaysia

## Abstract

In the title compound, C_15_H_14_N_2_O_3_, the dihedral angle between the benzene rings is 66.56 (5)°. In the crystal, N—H⋯O, O—H⋯O and C—H⋯O inter­actions link the mol­ecules into a three-dimensional network. A π–π inter­action, with a centroid–centroid distance of 3.628 (6) Å, helps to establish the packing.

## Related literature

For the properties of hydrazone derivatives, see: Lever (1972 ▶); Pelizzi & Pelizzi (1980 ▶). For related structures, see: Shan *et al.* (2003 ▶); Fun *et al.* (1996 ▶); Ferguson *et al.* (2005 ▶).
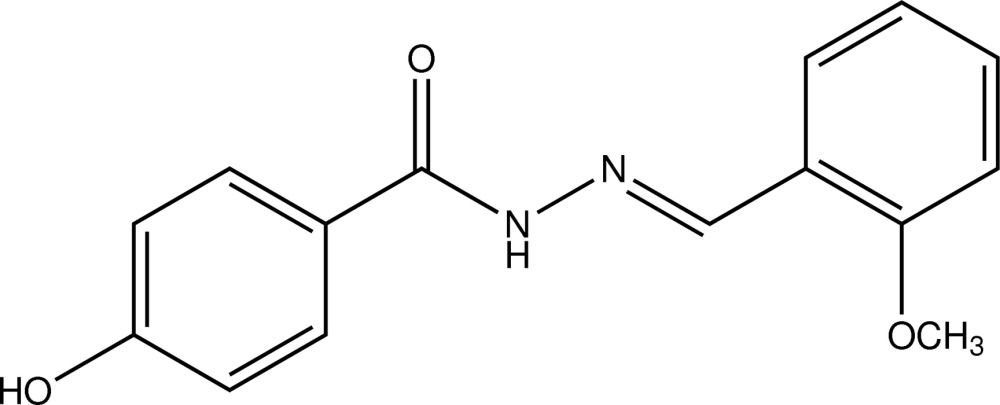


## Experimental

### 

#### Crystal data

C_15_H_14_N_2_O_3_
*M* *_r_* = 270.28
Orthorhombic, 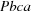

*a* = 14.3951 (3) Å
*b* = 8.7449 (2) Å
*c* = 21.1047 (4) Å
*V* = 2656.74 (10) Å^3^
*Z* = 8
Mo *K*α radiation
μ = 0.10 mm^−1^
*T* = 100 K
0.49 × 0.28 × 0.09 mm

#### Data collection

Bruker SMART APEXII CCD diffractometer
Absorption correction: multi-scan (*SADABS*; Bruker, 2009 ▶) *T*_min_ = 0.954, *T*_max_ = 0.991
32112 measured reflections
4442 independent reflections
3512 reflections with *I* > 2σ(*I*)
*R*_int_ = 0.036

#### Refinement

*R*[*F* ^2^ > 2σ(*F* ^2^)] = 0.044
*wR*(*F* ^2^) = 0.114
*S* = 1.03
4442 reflections
190 parameters
H atoms treated by a mixture of independent and constrained refinement
Δρ_max_ = 0.37 e Å^−3^
Δρ_min_ = −0.20 e Å^−3^

### 

Data collection: *APEX2* (Bruker, 2009 ▶); cell refinement: *SAINT* (Bruker, 2009 ▶); data reduction: *SAINT*; program(s) used to solve structure: *SHELXTL* (Sheldrick, 2008 ▶); program(s) used to refine structure: *SHELXTL*; molecular graphics: *SHELXTL*; software used to prepare material for publication: *SHELXTL* and *PLATON* (Spek, 2009 ▶).

## Supplementary Material

Crystal structure: contains datablock(s) I, global. DOI: 10.1107/S1600536812021952/hb6791sup1.cif

Structure factors: contains datablock(s) I. DOI: 10.1107/S1600536812021952/hb6791Isup2.hkl

Supplementary material file. DOI: 10.1107/S1600536812021952/hb6791Isup3.cml

Additional supplementary materials:  crystallographic information; 3D view; checkCIF report

## Figures and Tables

**Table 1:** Hydrogen-bond geometry (Å, °)

*D*—H⋯*A*	*D*—H	H⋯*A*	*D*⋯*A*	*D*—H⋯*A*
N1—H1*N*1⋯O2^i^	0.916 (16)	2.009 (16)	2.9202 (12)	172.8 (15)
O1—H1*O*1⋯O2^ii^	0.87 (2)	1.80 (2)	2.6528 (11)	164.2 (17)
C13—H13*A*⋯O1^iii^	0.95	2.52	3.4669 (15)	171

Symmetry codes: (i) 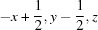; (ii) 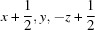; (iii) 
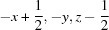
.

**Fig. 1.**
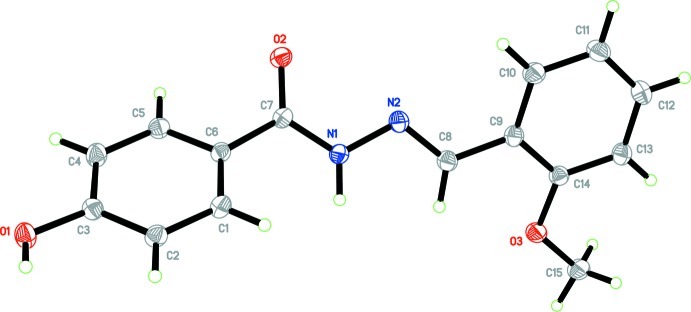
The molecular structure, showing 50% probability displacement ellipsoids. Hydrogen atoms are shown as spheres of arbitrary radius.

**Fig. 2.**
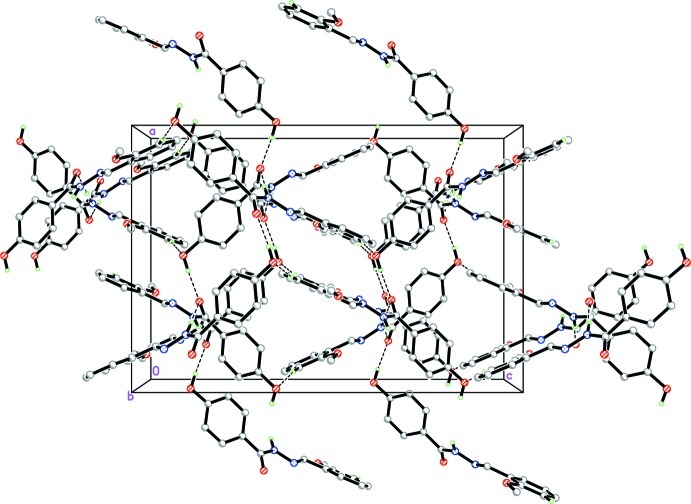
The crystal packing of (I). Dashed lines indicate hydrogen bonds. H atoms not involved in the hydrogen bond interactions have been omitted for clarity.

## supplementary crystallographic information

### Comment

The chemical properties of hydrazone derivatives have been intensively investigated in several research fields mainly due to their facile synthesis, tuneable electronic and steric properties, and good chelating ability (Pelizzi & Pelizzi, 1980). Some derivatives of the title compound, (I), were used for the determination of glucose (Lever, 1972). These compounds crystallize in the E conformation (Shan *et al.*, 2003; Fun *et al.*, 1996) and isomeric compounds have also been prepared (Ferguson *et al.*, 2005).

All parameters in (I), are within normal ranges. The dihedral angle between C1—C6 and C9 C14 benzene ring is 66.56 (5)°. In the crystal structure, the molecules are interconnected by N1—H1N1···O2^i^, O1—H1O1···O2^ii^ and C13—H13A···O1^iii^ hydrogen bonds. A π—π interaction with centroid-centroid distance of 3.628 (6) Å also occurs (*Cg*1 = C9—C14, -*x*, -*y*, -*z*).

### Experimental

A solution of 2-methoxybenzaldehyde (136 mg, 1 mmol) in methanol (10 ml) was added dropwise to a methanolic solution (10 ml) of 4-hydroxybenzhydrazide (152 mg, 1 mmol) and the mixture was refluxed for 2 h. The resulting solution was condensed on a steam bath to 5 ml and cooled to room temperature. Yellow plates were separated out, washed with cooled methanol and dried in air.

### Refinement

N and O bound H atoms were located in a difference Fourier map and freely refined. The remaining H atoms were positioned geometrically and refined using a riding model with C—H = 0.95–0.98 Å and *U*_iso_(H) = 1.2*U*_eq_(C) or 1.5*U*_eq_(C) for methyl H atoms. A rotating group model was applied to the methyl group.

### Crystal data

**Table:**

C_15_H_14_N_2_O_3_	*F*(000) = 1136
*M**_r_* = 270.28	*D*_x_ = 1.351 Mg m^−^^3^
Orthorhombic, *P**b**c**a*	Mo *K*α radiation, λ = 0.71073 Å
Hall symbol: -P 2ac 2ab	Cell parameters from 8771 reflections
*a* = 14.3951 (3) Å	θ = 2.4–31.5°
*b* = 8.7449 (2) Å	µ = 0.10 mm^−^^1^
*c* = 21.1047 (4) Å	*T* = 100 K
*V* = 2656.74 (10) Å^3^	Plate, yellow
*Z* = 8	0.49 × 0.28 × 0.09 mm

### Data collection

**Table:**

Bruker SMART APEXII CCD diffractometer	4442 independent reflections
Radiation source: fine-focus sealed tube	3512 reflections with *I* > 2σ(*I*)
Graphite monochromator	*R*_int_ = 0.036
φ and ω scans	θ_max_ = 31.6°, θ_min_ = 1.9°
Absorption correction: multi-scan (*SADABS*; Bruker, 2009)	*h* = −21→21
*T*_min_ = 0.954, *T*_max_ = 0.991	*k* = −12→12
32112 measured reflections	*l* = −30→31

### Refinement

**Table:**

Refinement on *F*^2^	Primary atom site location: structure-invariant direct methods
Least-squares matrix: full	Secondary atom site location: difference Fourier map
*R*[*F*^2^ > 2σ(*F*^2^)] = 0.044	Hydrogen site location: inferred from neighbouring sites
*wR*(*F*^2^) = 0.114	H atoms treated by a mixture of independent and constrained refinement
*S* = 1.03	*w* = 1/[σ^2^(*F*_o_^2^) + (0.0512*P*)^2^ + 0.959*P*] where *P* = (*F*_o_^2^ + 2*F*_c_^2^)/3
4442 reflections	(Δ/σ)_max_ < 0.001
190 parameters	Δρ_max_ = 0.37 e Å^−^^3^
0 restraints	Δρ_min_ = −0.20 e Å^−^^3^

### Special details

**Table:**

Experimental. The crystal was placed in the cold stream of an Oxford Cryosystems Cobra open-flow nitrogen cryostat (Cosier & Glazer, 1986) operating at 100.0 (1) K.
Geometry. All e.s.d.'s (except the e.s.d. in the dihedral angle between two l.s. planes) are estimated using the full covariance matrix. The cell e.s.d.'s are taken into account individually in the estimation of e.s.d.'s in distances, angles and torsion angles; correlations between e.s.d.'s in cell parameters are only used when they are defined by crystal symmetry. An approximate (isotropic) treatment of cell e.s.d.'s is used for estimating e.s.d.'s involving l.s. planes.
Refinement. Refinement of *F*^2^ against ALL reflections. The weighted *R*-factor *wR* and goodness of fit *S* are based on *F*^2^, conventional *R*-factors *R* are based on *F*, with *F* set to zero for negative *F*^2^. The threshold expression of *F*^2^ > σ(*F*^2^) is used only for calculating *R*-factors(gt) *etc*. and is not relevant to the choice of reflections for refinement. *R*-factors based on *F*^2^ are statistically about twice as large as those based on *F*, and *R*- factors based on ALL data will be even larger.

### Fractional atomic coordinates and isotropic or equivalent isotropic displacement parameters (Å^2^)

**Table:**

	*x*	*y*	*z*	*U*_iso_*/*U*_eq_	
O1	0.48726 (6)	0.48027 (10)	0.36875 (4)	0.02079 (17)	
O2	0.16105 (5)	0.45718 (9)	0.17195 (4)	0.01884 (16)	
O3	0.13520 (6)	−0.20688 (9)	0.02729 (4)	0.02154 (18)	
N1	0.22355 (7)	0.22544 (11)	0.14758 (4)	0.01752 (18)	
N2	0.17102 (6)	0.21485 (11)	0.09246 (4)	0.01848 (19)	
C1	0.38142 (7)	0.31733 (13)	0.22565 (5)	0.0181 (2)	
H1A	0.3970	0.2544	0.1905	0.022*	
C2	0.44845 (7)	0.35161 (13)	0.27091 (5)	0.0184 (2)	
H2A	0.5098	0.3130	0.2665	0.022*	
C3	0.42528 (7)	0.44303 (13)	0.32286 (5)	0.0170 (2)	
C4	0.33481 (8)	0.49850 (14)	0.32950 (5)	0.0204 (2)	
H4A	0.3185	0.5583	0.3654	0.024*	
C5	0.26913 (7)	0.46623 (13)	0.28374 (5)	0.0187 (2)	
H5A	0.2082	0.5066	0.2878	0.022*	
C6	0.29126 (7)	0.37485 (12)	0.23154 (5)	0.01578 (19)	
C7	0.21981 (7)	0.35457 (12)	0.18194 (5)	0.01583 (19)	
C8	0.17442 (7)	0.08270 (13)	0.06597 (5)	0.0185 (2)	
H8A	0.2048	0.0008	0.0871	0.022*	
C9	0.13238 (7)	0.05622 (13)	0.00393 (5)	0.0176 (2)	
C10	0.11156 (8)	0.17623 (13)	−0.03728 (5)	0.0210 (2)	
H10A	0.1240	0.2785	−0.0246	0.025*	
C11	0.07298 (8)	0.14849 (14)	−0.09648 (5)	0.0229 (2)	
H11A	0.0582	0.2310	−0.1240	0.027*	
C12	0.05632 (8)	−0.00132 (15)	−0.11500 (5)	0.0227 (2)	
H12A	0.0301	−0.0208	−0.1555	0.027*	
C13	0.07724 (8)	−0.12288 (14)	−0.07539 (5)	0.0209 (2)	
H13A	0.0659	−0.2248	−0.0889	0.025*	
C14	0.11498 (7)	−0.09490 (13)	−0.01561 (5)	0.0176 (2)	
C15	0.11450 (9)	−0.36068 (13)	0.00934 (6)	0.0237 (2)	
H15A	0.1294	−0.4297	0.0445	0.036*	
H15B	0.1516	−0.3886	−0.0278	0.036*	
H15C	0.0483	−0.3692	−0.0009	0.036*	
H1N1	0.2628 (11)	0.1465 (19)	0.1575 (7)	0.027 (4)*	
H1O1	0.5437 (14)	0.455 (2)	0.3577 (9)	0.050 (5)*	

### Atomic displacement parameters (Å^2^)

**Table:**

	*U*^11^	*U*^22^	*U*^33^	*U*^12^	*U*^13^	*U*^23^
O1	0.0171 (4)	0.0257 (4)	0.0195 (3)	−0.0008 (3)	−0.0020 (3)	−0.0037 (3)
O2	0.0166 (3)	0.0165 (4)	0.0233 (4)	0.0005 (3)	−0.0012 (3)	−0.0014 (3)
O3	0.0272 (4)	0.0151 (4)	0.0223 (4)	−0.0010 (3)	−0.0063 (3)	0.0006 (3)
N1	0.0184 (4)	0.0148 (4)	0.0194 (4)	−0.0003 (4)	−0.0037 (3)	−0.0015 (3)
N2	0.0178 (4)	0.0190 (4)	0.0187 (4)	−0.0013 (4)	−0.0023 (3)	−0.0012 (3)
C1	0.0176 (5)	0.0176 (5)	0.0192 (4)	−0.0007 (4)	0.0017 (3)	−0.0031 (4)
C2	0.0152 (4)	0.0187 (5)	0.0213 (4)	0.0012 (4)	0.0011 (3)	−0.0024 (4)
C3	0.0169 (4)	0.0167 (5)	0.0174 (4)	−0.0022 (4)	−0.0004 (3)	0.0007 (4)
C4	0.0199 (5)	0.0234 (5)	0.0178 (4)	0.0015 (5)	0.0011 (4)	−0.0039 (4)
C5	0.0169 (4)	0.0201 (5)	0.0191 (4)	0.0021 (4)	0.0010 (4)	−0.0018 (4)
C6	0.0158 (4)	0.0140 (4)	0.0175 (4)	−0.0009 (4)	0.0002 (3)	−0.0005 (4)
C7	0.0153 (4)	0.0146 (5)	0.0175 (4)	−0.0023 (4)	0.0021 (3)	−0.0003 (4)
C8	0.0185 (5)	0.0175 (5)	0.0196 (4)	−0.0014 (4)	−0.0022 (3)	0.0001 (4)
C9	0.0168 (4)	0.0180 (5)	0.0180 (4)	−0.0020 (4)	−0.0007 (3)	−0.0014 (4)
C10	0.0233 (5)	0.0169 (5)	0.0229 (5)	−0.0019 (4)	−0.0017 (4)	0.0007 (4)
C11	0.0258 (5)	0.0215 (5)	0.0213 (5)	−0.0004 (5)	−0.0023 (4)	0.0043 (4)
C12	0.0233 (5)	0.0261 (6)	0.0186 (4)	−0.0013 (5)	−0.0031 (4)	−0.0002 (4)
C13	0.0230 (5)	0.0195 (5)	0.0201 (4)	−0.0016 (5)	−0.0027 (4)	−0.0026 (4)
C14	0.0173 (4)	0.0172 (5)	0.0183 (4)	0.0000 (4)	−0.0008 (3)	−0.0001 (4)
C15	0.0288 (6)	0.0146 (5)	0.0278 (5)	−0.0019 (5)	−0.0038 (4)	−0.0006 (4)

### Geometric parameters (Å, º)

**Table:**

O1—C3	1.3565 (12)	C5—H5A	0.9500
O1—H1O1	0.87 (2)	C6—C7	1.4782 (14)
O2—C7	1.2510 (13)	C8—C9	1.4609 (14)
O3—C14	1.3650 (13)	C8—H8A	0.9500
O3—C15	1.4287 (14)	C9—C10	1.3956 (15)
N1—C7	1.3432 (14)	C9—C14	1.4068 (16)
N1—N2	1.3905 (12)	C10—C11	1.3885 (15)
N1—H1N1	0.917 (16)	C10—H10A	0.9500
N2—C8	1.2848 (14)	C11—C12	1.3880 (17)
C1—C2	1.3904 (14)	C11—H11A	0.9500
C1—C6	1.3974 (15)	C12—C13	1.3856 (16)
C1—H1A	0.9500	C12—H12A	0.9500
C2—C3	1.3973 (15)	C13—C14	1.3953 (14)
C2—H2A	0.9500	C13—H13A	0.9500
C3—C4	1.3969 (15)	C15—H15A	0.9800
C4—C5	1.3806 (15)	C15—H15B	0.9800
C4—H4A	0.9500	C15—H15C	0.9800
C5—C6	1.3978 (14)		
			
C3—O1—H1O1	111.1 (12)	N2—C8—C9	121.12 (10)
C14—O3—C15	117.06 (8)	N2—C8—H8A	119.4
C7—N1—N2	119.07 (9)	C9—C8—H8A	119.4
C7—N1—H1N1	122.3 (9)	C10—C9—C14	119.05 (10)
N2—N1—H1N1	118.5 (9)	C10—C9—C8	121.89 (10)
C8—N2—N1	113.78 (9)	C14—C9—C8	119.03 (10)
C2—C1—C6	120.38 (10)	C11—C10—C9	121.01 (11)
C2—C1—H1A	119.8	C11—C10—H10A	119.5
C6—C1—H1A	119.8	C9—C10—H10A	119.5
C1—C2—C3	119.79 (10)	C12—C11—C10	119.17 (10)
C1—C2—H2A	120.1	C12—C11—H11A	120.4
C3—C2—H2A	120.1	C10—C11—H11A	120.4
O1—C3—C4	117.28 (9)	C13—C12—C11	121.11 (10)
O1—C3—C2	122.73 (10)	C13—C12—H12A	119.4
C4—C3—C2	119.99 (10)	C11—C12—H12A	119.4
C5—C4—C3	119.82 (10)	C12—C13—C14	119.72 (11)
C5—C4—H4A	120.1	C12—C13—H13A	120.1
C3—C4—H4A	120.1	C14—C13—H13A	120.1
C4—C5—C6	120.81 (10)	O3—C14—C13	123.85 (10)
C4—C5—H5A	119.6	O3—C14—C9	116.20 (9)
C6—C5—H5A	119.6	C13—C14—C9	119.93 (10)
C1—C6—C5	119.18 (9)	O3—C15—H15A	109.5
C1—C6—C7	122.68 (9)	O3—C15—H15B	109.5
C5—C6—C7	117.92 (9)	H15A—C15—H15B	109.5
O2—C7—N1	122.63 (9)	O3—C15—H15C	109.5
O2—C7—C6	120.24 (9)	H15A—C15—H15C	109.5
N1—C7—C6	117.09 (9)	H15B—C15—H15C	109.5
			
C7—N1—N2—C8	−175.07 (10)	N1—N2—C8—C9	−172.79 (9)
C6—C1—C2—C3	0.55 (17)	N2—C8—C9—C10	19.20 (16)
C1—C2—C3—O1	179.77 (10)	N2—C8—C9—C14	−162.77 (10)
C1—C2—C3—C4	0.59 (17)	C14—C9—C10—C11	0.93 (17)
O1—C3—C4—C5	179.00 (10)	C8—C9—C10—C11	178.95 (10)
C2—C3—C4—C5	−1.77 (17)	C9—C10—C11—C12	−0.96 (18)
C3—C4—C5—C6	1.84 (17)	C10—C11—C12—C13	0.22 (18)
C2—C1—C6—C5	−0.50 (16)	C11—C12—C13—C14	0.54 (18)
C2—C1—C6—C7	173.99 (10)	C15—O3—C14—C13	−1.07 (16)
C4—C5—C6—C1	−0.70 (16)	C15—O3—C14—C9	177.53 (10)
C4—C5—C6—C7	−175.45 (10)	C12—C13—C14—O3	177.98 (10)
N2—N1—C7—O2	10.90 (15)	C12—C13—C14—C9	−0.57 (16)
N2—N1—C7—C6	−166.78 (9)	C10—C9—C14—O3	−178.81 (10)
C1—C6—C7—O2	−145.49 (11)	C8—C9—C14—O3	3.10 (14)
C5—C6—C7—O2	29.07 (15)	C10—C9—C14—C13	−0.15 (16)
C1—C6—C7—N1	32.25 (15)	C8—C9—C14—C13	−178.24 (10)
C5—C6—C7—N1	−153.19 (10)		

### Hydrogen-bond geometry (Å, º)

**Table:**

*D*—H···*A*	*D*—H	H···*A*	*D*···*A*	*D*—H···*A*
N1—H1*N*1···O2^i^	0.916 (16)	2.009 (16)	2.9202 (12)	172.8 (15)
O1—H1*O*1···O2^ii^	0.87 (2)	1.80 (2)	2.6528 (11)	164.2 (17)
C13—H13*A*···O1^iii^	0.95	2.52	3.4669 (15)	171

Symmetry codes: (i) −*x*+1/2, *y*−1/2, *z*; (ii) *x*+1/2, *y*, −*z*+1/2; (iii) −*x*+1/2, −*y*, *z*−1/2.
